# Overview of next-generation sequencing to the molecular diagnosis of inborn errors of immunity in Brazil: a systematic review

**DOI:** 10.3389/fimmu.2026.1794921

**Published:** 2026-05-15

**Authors:** Filipe Vicente dos Santos-Bueno, Elissa Santos Morgado, Bruna Nunes da Silva Agonigi, Milena Regina Gomes Lopes, Marcella de Oliveira Pinheiro, Luciene Lima da Silva, Margarida dos Santos Salú, Roberta Soares Faccion, Zilton Farias Meira de Vasconcelos

**Affiliations:** 1 Laboratorio de Alta Complexidade (LACIFF), Unidade de Pesquisa Clinica, Instituto Nacional de Saude da Mulher, da Crianca e do Adolescente Fernandes Figueira (IFF), Fundacao Oswaldo Cruz (Fiocruz), Rio de Janeiro, Brazil; 2 Programa de Pós-graduação em Pesquisa Aplicada à Saúde da Criança e da Mulher, Instituto Nacional de Saúde da Mulher, da Criança e do Adolescente Fernandes Figueira (IFF), Fundação Oswaldo Cruz (Fiocruz), Rio de Janeiro, Brazil; 3 Laboratório de Imunofisiologia, Instituto de Biofísica Carlos Chagas Filho, Universidade Federal do Rio de Janeiro, Rio de Janeiro, Brazil

**Keywords:** Brazil, exome sequencing, genetic sequencing, inborn errors of immunity, low- and middle-income country, next-generation sequencing, whole exome sequencing

## Abstract

**Background:**

Inborn errors of immunity (IEI) are rare monogenic disorders affecting the immune system, leading to immunodeficiency, autoinflammation, autoimmunity, allergy, and/or malignancy. The identification of IEI has accelerated with next-generation sequencing (NGS), increasing the molecular diagnostic rate. In Latin America, IEI prevalence is about 1 in 10, 000 individuals. However, the limited availability of NGS in Brazil delays early diagnosis, personalized therapeutic interventions, and comprehensive genetic counseling. To our knowledge no systematic review has yet aimed to summarize the Brazilian studies employing NGS methodologies for the genetic diagnosis of IEI.

**Methods:**

This review was conducted in accordance with PRISMA guidelines and is registered with PROSPERO (CRD420251059458). A comprehensive systematic search of Embase, MEDLINE, SciELO and LILACS databases was performed to identify studies published up to April 2025. Inclusion criteria were restricted to Brazilian studies reporting on native patients diagnosed with IEI that employed NGS methodologies.

**Results:**

From 242 studies initially found, 10 studies filled the inclusion criteria, comprising six case reports and four case series, reporting data from 419 patients, of whom 82 were diagnosed with IEI (19.6%), the majority of whom were diagnosed with SCID, CGD, and XLA. The studies were conducted in the Southeast, Northeast, and South regions of Brazil, which evidences a lack of studies in the North and Middle-West Brazilian regions. Considerable variability was observed among studies in reporting methodological details of NGS workflows, including sequencing platforms, bioinformatics pipelines, and quality control metrics. Although strategies based on WES predominated, platform specifications were often incomplete or omitted. Bioinformatic workflows were inconsistently detailed, with variable reporting of alignment tools, reference genomes, variant callers, and annotation software. Quality control metrics and thresholds lack standardization, limiting comparability. Variant prioritization and interpretation approaches varied, though ACMG/AMP guidelines were generally followed when reported, highlighting the need for standardized, transparent pipelines to improve reproducibility and diagnostic accuracy.

**Conclusion:**

The findings of this systematic review reveal few Brazilian studies employing NGS for IEI diagnosis. Limited utilization hinders early subtype identification and clinical management of patients. Large multicentric studies covering all regions and standardized reporting are needed to inventory IEI epidemiology and guide national diagnosis policies.

**Systematic review registration:**

https://www.crd.york.ac.uk/prospero/, identifier CRD420251059458.

## 1 Introduction

Human inborn errors of immunity (IEI) constitute a diverse group of monogenic disorders that disrupt both innate and adaptive immune responses, as well as immune regulation. These conditions exhibit various inheritance patterns, including autosomal dominant, autosomal recessive, and X-linked modes, with diverse clinical phenotypes that may display complete or partial penetrance. Individuals affected by IEI commonly show heightened vulnerability to a wide range of infectious agents, increased risk of malignancies, which is usually associated with loss-of-function (LOF) mutations, alongside manifestations such as autoimmunity, autoinflammation, and allergic reactions, are more related to gain-of-function (GOF) genetic alterations (1).

Currently, various DNA sequencing methodologies are employed to identify mutations associated with diseases. Prior to 2010, Sanger sequencing constituted the primary approach for routine genetic testing in clinical practice (2). The identification of IEI has accelerated dramatically with the advent of next-generation sequencing (NGS), revealing not only rare, but also more prevalent genetic variants (3, 4). Furthermore, NGS has substantially transformed the management of IEI, as genotypic characterization has facilitated a deeper understanding of the immunopathogenesis of IEI, enabling the development of targeted therapeutic protocols that enhance overall survival and improve quality of life in affected individuals (5, 6). According to the most recent update by the International Union of Immunological Societies (IUIS) expert committee, the last few decades have seen the identification of more than 500 genes causing IEI. Before NSG strategies gained place in clinical practice, the estimated prevalence of IEI in Latin America was about 1 case per 10, 000 individuals, while more recent data estimates the global cumulative prevalence to be between around 1 in 1, 000 and 1 in 5, 000 individuals (7, 8).

In Brazil, the implementation of NGS in clinical and research settings has progressively increased, reflecting global trends toward precision medicine (9, 10). The Brazilian Policy for Comprehensive Care for Persons with Rare Diseases (Portaria GM/MS n° 199/2014) established reference services and specialized care networks within Unified Health System (SUS), with most services of reference and specialized attention concentrated in the Southeast and Northeast regions (11). Complementing this framework, initiatives such as the *Genomas Brasil* National Program advanced precision medicine through genomic sequencing for enhanced diagnostic accuracy and disease risk prediction, while the Rare Genomes Project was the first whole-genome sequencing (WGS) project for rare diseases in the country (12). In 2020, the Brazilian Rare Diseases Network (RARAS) emerged, uniting University Hospitals, Neonatal Screening Services, and Rare Diseases Reference Centers to bridge NGS implementation gaps (11). More recently, the National Genomics Network for Inborn Errors of Immunity (RENOMIEII) — a pioneering project established in 2023 with support from the Department of Science and Technology (DECIT) and coordinated by the National Institute of Women’s, Children’s, and Adolescents’ Health Fernandes Figueira (IFF/Fiocruz) — further strengthens these efforts by integrating SUS healthcare institutions and academic research centers. RENOMIEII employs various omics methodologies (genomics, proteomics, metabolomics, and single-cell transcriptomics) to assess their impact on the prognosis, treatment, and genetic counseling of patients with IEI. These collaborative efforts have fostered integration among genetics and bioinformatics centers nationwide, facilitating clinical genomic incorporation into SUS.

Despite these advances, the adoption of NGS for diagnosing IEI in Brazil continues to face major challenges. Variations in sequencing platforms, analytical pipelines, and reporting standards pose challenges to data reproducibility and inter-laboratory comparability. Furthermore, limited representation of the Brazilian population in international genomic databases hinders the interpretation of variants of uncertain significance (VUS), emphasizing the need for local genomic reference data. Variant classification is often based on population frequency data from widely used resources such as gnomAD, ExAC, and the 1000 Genomes Project, which are predominantly composed of individuals of European ancestry. This imbalance may lead to misclassification of variants in highly admixed populations such as Brazil, increasing the likelihood of inconclusive or inaccurate interpretations. Two genomic databases currently support precision medicine in Brazil: ABraOM (abraom.ib.usp.br), a public repository of variants from 1, 171 elderly individuals enabling RefSeq-annotated gene/region queries for pathogenicity assessment in admixed populations; and the DNA do Brasil project (12), which encompasses urban, rural, and indigenous cohorts to integrate genomic data into SUS for disease risk prediction. These initiatives enhance rare disease diagnostics and precision medicine implementation (13).

Over the past decade, the global adoption of NGS has transformed the diagnostic landscape for rare and immune-related disorders. Integrated whole exome sequencing (WES) and whole genome sequencing (WGS) into clinical practice leads to early diagnosis, improved disease classification, and targeted therapeutic interventions. These programs have demonstrated that comprehensive genomic testing can significantly reduce diagnostic delays and improve clinical outcomes for patients with IEI (14). However, the incorporation of NGS into healthcare systems varies widely across countries, reflecting differences in infrastructure, funding models, and population genomics representation.

In this context, the present systematic review aims to map and analyze studies employing NGS for the diagnosis of IEI in Brazil, summarizing their clinical and methodological characteristics, identifying gaps in standardization, and highlighting opportunities for future genomic integration into national healthcare and research networks.

## 2 Methods

A systematic review of the literature was conducted in accordance with the Preferred Reporting Items for Systematic Reviews and Meta-Analyses (PRISMA) guidelines, with the objective of summarizing Brazilian studies that employed NGS methodologies for the genetic diagnosis of IEI (15). The study protocol was registered with PROSPERO (registration number CRD420251059458) (16).

### 2.1 Data source and search strategy

An extensive literature search was conducted using PubMed, Embase, SciELO, LILACs, and Scopus databases up to April 7, 2025, with no language restrictions. Initial searches using MeSH terms (“Primary Immunodeficiency Diseases”, “Whole Genome Sequencing”, “Exome Sequencing”, “Brazil”) yielded insufficient relevant articles, with most retrieved records falling outside the systematic review’s scope. Consequently, terms searched in Title/Abstract fields — “inborn errors of immunity”, “primary immunodeficiency”, “whole-genome sequencing”, “exome sequencing”, “gene sequencing panel”, “genetic sequencing”, and “Brazil” — were employed to comprehensively capture studies on NGS applications for IEI diagnosis in Brazil.

### 2.2 Inclusion and exclusion criteria

The inclusion criteria for this review comprised original research articles that involved patients diagnosed with inborn errors of immunity (IEI), including case series and case reports, and that applied next-generation sequencing (NGS) methodologies for genetic diagnosis.

The exclusion criteria included abstracts published in conferences, meetings, guidelines not indexed in scientific journals, studies employing methodologies other than NGS for the diagnosis of IEI, and review articles.

### 2.3 Data extraction and risk of bias assessment

Data extraction was initially performed individually by one of the author’s (FB) and included the following steps (1): identification of studies reporting inborn errors of immunity (IEI) and (2) selection of Brazilian studies meeting the predefined inclusion criteria. The EndNote Web software (https://www.myendnoteweb.com/) was used to manage references and remove duplicates. Subsequently, a team of reviewers (EM, BA, ML, MP, LL, MS, RF, and ZV) examined the selected articles to extract relevant data—including study and patient geographic location, total case numbers, identified IEI subtypes, NGS methodology, sequencing platform, reference genome, bioinformatics pipeline, quality control metrics, allelic frequency estimation, pathogenicity predictors, variant prioritization approach, clinical interpretation tools, cases with confirmed genetic diagnosis, other clinical manifestations, infection history, and affected genes — for further analysis. Manual data extraction was also performed by the authors, and potential confounding factors were evaluated collectively by all team members. When technical sequencing details were not available in the original publications, additional information was retrieved from publicly available sources (e.g., institutional or commercial websites such as 3Billion – **Table 1**).

**Table 1:** Overview of NGS data processing, quality control metrics, variant filtering and clinical interpretation across studies.

First Author, Year	NGS method	Sequencing platform (Library/Enrichment)	Reference genome	Bioinformatics pipeline*	Quality control metrics	Allelic frequency estimation	Pathogenicity predictors	Variant prioritization approach	Clinical interpretation tools
Napoleao, SMS et al, 2025 ​​ (17)	WES	Illumina HiSeq2000 (Illumina TruSeq library/Agilent SureSelect Human All Exon)	GRCh37/hg19	SAMtools and GATK	NR	MAF	CADD, PolyPhen2, SIFT and MutationTaster**	•IUIS gene list (2022) •MAF ≤1% •CADD score > 15	NR
Sbruzzi, RC et al, 2024 ​ (18) ​	WES	NR	NR	NR	NR	MAF	CADD, MAPP, AlphaMissense, SIFT, PolyPhen2, MutationTaster, MutPred2 and Consurf	•Homozigous SNV (nonssense, missense or splice site alterations) and INDEL •MAF ≤1% •CADD score > gene’s MSC	•ACMG/AMP criteria
Prestes-Carneiro, LE et al, 2023 ​ (19)	Targeted gene panel (74 genes)	Illumina HiSeq (Illumina Nextera Rapid Capture Mendelics Custom Panel V2)	GRCh37/hg19	NR	NR	NR	NR	NR	NR
	WES	Illumina NovaSeq X^†^ (3B-Exome report by 3Billion)	NR	NR	•Mean depth of coverage ~100x^†^ •98% of targeted regions covered at ≥20×^†^	NR	NR	NR	NR
Francisco Junior, RS et al, 2022 ​​ (20)	WES	Illumina NextSeq^®^ 500/550^‡^ ^±^ (Illumina TruSeq library) ^‡^	GRCh38/hg38^‡^	Bowtie2, SAMtools, Picard, GATK UnifiedGenotyper and SnpEff/SnpSift^‡^	•Base quality score > 30 (Q30)^‡^ •96% of exonic regions covered at ≥100×	MAF based on population databases (GnomAD, dbSNP, ExAC and 1000Genomes)	BayesDel_addAF, CADD, DEOGEN2, FATHMM-MKL, LIST-S2, M-CAP, MVP, Mutation-Assessor, MutationTaster, Polyphen2-HVAR, PrimateAI and SIFT	•Phenotype-associated gene list (reported in HPO and HGMD databases) •SNV and INDEL	•VarSome − variant classification based on ACMG/AMP criteria •HLAminer – HLA haplotype prediction used to correlate HLA type with patient phenotype (HPO terms) ^‡^
Mendonça, LO et al, 2020 ​​ (21)	Targeted gene panel (10 genes - SAID)	Ion PGM™ (Ion AmpliSeq designer)	NR	NR	NR	NR	NR	NR	NR
Freire, BL et al, 2017 ​ (22)	WES	Illumina HiSeq 2500 (Agilent SureSelect Human All Exon V6)	GRCh37/hg19	BWA-MEM, Biobambam2, Freebaeys and ANNOVAR	•Average depth of coverage ~122x •98.5% of targeted exonic regions covered at ≥10×	MAF based on in-house sequencing data sets and databases (GnomAD, ABraOM, and 1000Genomes)	CADD, PolyPhen2, SIFT, PROVEAN and Mutation-Assessor^‡^	•Homozigous non-synonymous SNV (nonssense, missense or splice-site alterations) •MAF ≤1%	•OMIM and PubMed databases - assessment of gene function •ACMG/AMP criteria
Ferreira, CS et al, 2023 ​​ (9)​	WES	Illumina NextSeq^®^ 500/550 ^±^ (Illumina TruSeq library)	GRCh38/hg38	Bowtie2, SAMtools, Picard, GATK HaplotypeCaller and SnpEff/SnpSift^‡^	•Base quality score > 30 (Q30) •Depth of coverage (per exon) > 122x^‡^ •~92% of exonic bases covered at ≥20×^‡^	MAF	NR	•Protein altering SNV and INDEL •MAF ≤1% •Pathogenic and Likely pathogenic •Clinical phenotype based on HPO terms • IUIS gene list (2022)	•VarSome - variant classification based on ACMG/AMP criteria •Franklin (Genoox) - variant filtering and selection based on phenotype (HPO term) and gene list. •OMIM - gene enheritance pattern
Ferreira, CS et al, 2023 ​​ (10)	WES	Illumina NextSeq^®^ 500/550 ^±^ (Illumina TruSeq library)	GRCh38/hg38	Bowtie2, SAMtools, Picard, GATK HaplotypeCaller, SnpEff/SnpSift and VEP	•Base quality score > 30 (Q30) •Depth of coverage (per exon) > 120x^‡^ •~92% of exonic bases covered at ≥20×^‡^	MAF based on databases (GnomAD, 1000Genomes, and ExAC)	SIFT, PolyPhen, CADD and LoFtool (tools score consulted on VEP)	•Protein altering SNV and INDEL •MAF ≤1% •Pathogenic and Likely pathogenic •Clinical phenotype based on HPO terms •IUIS gene list (2022)	•VarSome - variant classification based on ACMG/AMP criteria •Franklin (Genoox) - variant filtering and selection based on phenotype (HPO term) and gene list. •OMIM - gene enheritance pattern
Lyra, PT et al, 2022 ​​ (23)	Targeted gene panel (361 genes - IEI)	Illumina Next-Seq^®^ (Agilent ClearSeq Inherited Disease panel)	NR	GATK and ANNOVAR	•≥90% of bases with quality score > 30 (Q30) •Mapped reads > 98% and coverage uniformity ≥ 80%. •20× minimum coverage across whole genome and autosomes^§^	NR	NR	NR	NR
Quaio, CRDC et al, 2021 ​​ (24)	WES	Illumina Next-Seq^®^ (Agilent Clinical Research Exome v1)^¶^	GRCh37/hg19^¶^	BWA-MEM, GATK and VEP^¶^	•≥95% of targeted bases covered at > 10×^¶^	MAF	NR	•Non-synonymous SNV and INDEL^¶^ •Association with recessive conditions reported on clinical databases (ClinVar and HGMD) and literature; OR MAF < 1%, *in silico* prediction of functional impact and located in clinically relevant gene •Pathogenic and Likely pathogenic	•In-house Web interface OR EmedGene were used for variant analysis and clinical interpretation^¶^ •VarSome - variant classification based on ACMG/AMP criteria • Genes grouped as “Immune disorders” based on IUIS 2019

* Pipeline roles of softwares/tools: Aligner — BWA-MEM, Bowtie2; Post-alignment processing — SAMtools, Picard, Biobambam2; Variant caller — GATK HaplotypeCaller, GATK UnifiedGenotyper, FreeBayes; Variant annotation — ANNOVAR, VEP, SnpEff/SnpSift (annotation + filtering).

** PolyPhen-2, SIFT, and MutationTaster were mentioned in the results, but it is unclear whether they were used directly by the authors or merely cited from the literature.

† Technical details of 3B-Exome Report (3Billion, Republic of South Korea) used for WES not provided. Data was obtained directly from company’s website (https://3billion.io/whole-exome-sequencing).

‡ Data extracted from supplementary material.

± Information inferred from the sequencing kit “Illumina NextSeq^®^ 500/550 High Output V2”, though is insufficient to determined which system (NextSeq 500 or 550) was used.

§ Data reported on manuscript, although the sequencing method used was a *targeted 361-gene panel*. Thus, this value refers to coverage depth of the targeted regions, not full genome or autosome coverage.

¶ Data from Quaio CRDC et al, 2020 (25) that was explicitly cited by Quaio, CRDC et al, 2021 (24) for this methods section, since the previous manuscript encompasses the full cohort submitted to WES. ABraOM, Brazilian genomic variants database; ACMG/AMP, American College of Medical Genetics and Genomics/Association for Molecular Pathology; dbSNP, Single Nucleotide Polymorphism Database; ExAC, Exome Aggregation Consortium; GnomAD, Genome Aggregation Database; HGMD, Human Gene Mutation Database; HLA, Human Leukocyte Antigen; HPO, Human Phenotype Ontology; IEI, Inborn Errors of Immunity; INDEL, Insertion/deletion; IUIS, International Union of Immunological Societies; MAF, Minor allele frequency; MSC, Mutation Significance Cutoff; NGS, New Generation Sequencing; NR, Not reported; OMIM, Online Mendelian Inheritance in Man; Q30, Phred quality score ≥30; SAID, Systemic Auto-Inflammatory Diseases; SNV, Single-nucleotide variant; WES, Whole-exome sequencing.

The methodological quality and risk of bias of included studies were assessed using Joanna Briggs Institute (JBI) critical appraisal tools (26), comprising 8 items for case reports (patient demographics/history, clinical presentation, diagnostics/treatment, post-intervention outcomes, adverse events, and takeaway lessons) and 10 items for case series (inclusion criteria, measurement/identification methods, consecutive/complete enrollment, demographics/clinical data, outcomes/follow-up, site information, and statistical analysis). Each item was rated as “yes” (met), “no, “ “unclear, “ or “not applicable.” Studies were stratified by total score into high (<5), moderate (5, 6), or low (≥7) bias risk. Two independent reviewers (FVSB, BA) performed assessments, resolving discrepancies via discussion with a third reviewer (EM). Detailed results are presented in **Supplementary Table 1**.

## 3 Results

### 3.1 Characteristics of included studies in this systematic literature review

**Figure 1:**
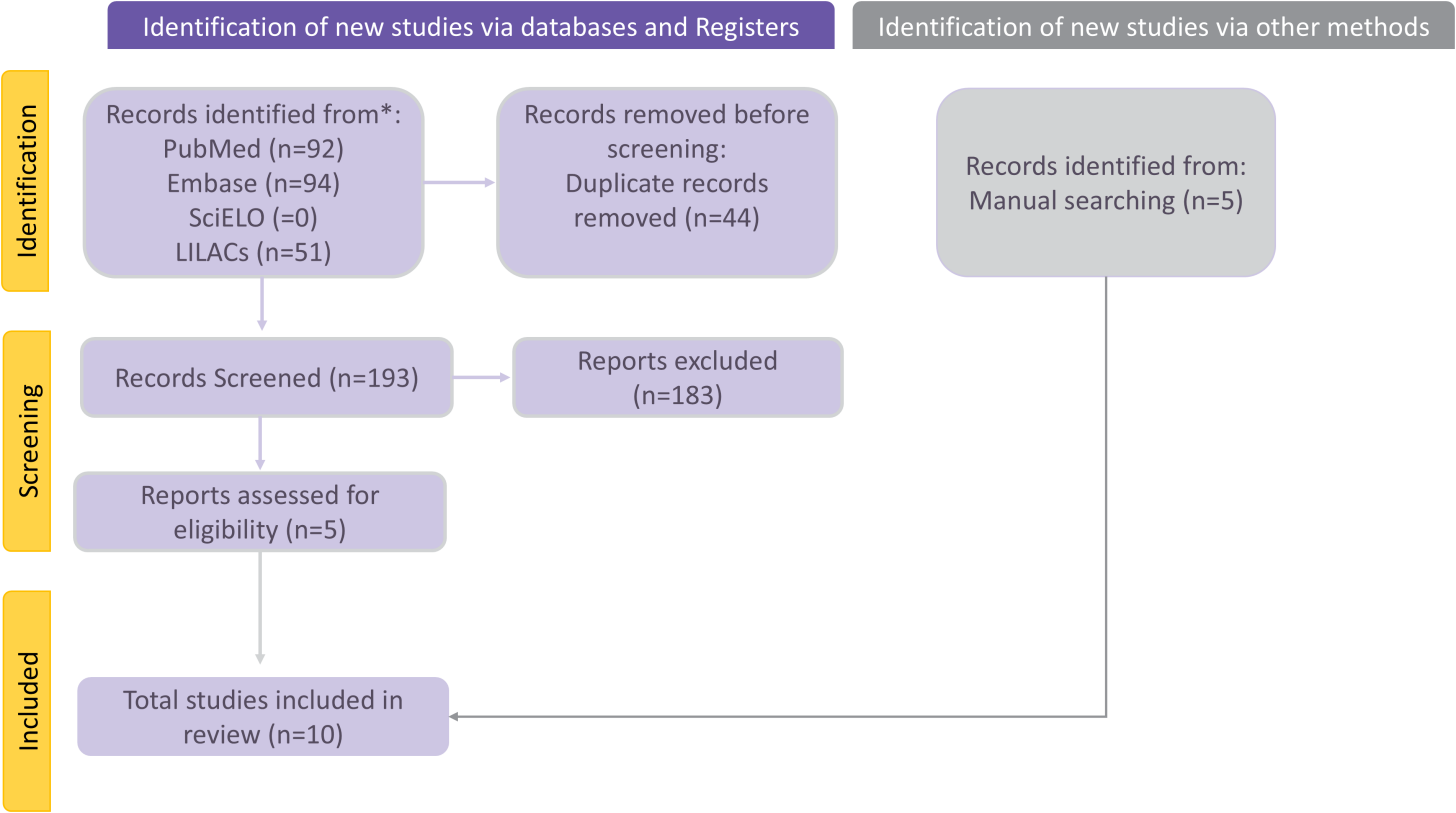
Flow diagram of study selection process adapted from PRISMA flow chart (15). The search strategy initially identified 237 records across multiple databases (EMBASE: 94; MEDLINE: 92; SciELO: 0 and LILACS: 51). Following duplicate removal, 44 unique studies were retained for screening. Title and abstract evaluation led to the exclusion of 183 articles irrelevant to the research focus. Database searches yielded five eligible studies, complemented by an additional five studies identified through manual searches. Consequently, a total of ten studies fulfilled the inclusion criteria and were incorporated into the review.

The search strategy retrieved a total of 237 records from all databases (EMBASE: 94; MEDLINE: 92; SciELO: 0; LILACS: 51). After removing duplicates, 44 unique studies remained. Titles and abstracts were screened, resulting in the exclusion of 183 studies that did not address the research topic. Excluded studies included non-human studies, studies not involving IEI, studies not using NGS methodologies, and review articles. Five studies (9, 10, 17, 19, 24) were identified through database searches, and an additional five (18, 20–23) were retrieved through manual search. In total, ten studies (9, 10, 17–24) met the eligibility criteria and were included in the review (**Figure 1**). These studies were published between 2017 and 2024. Six were case reports (17–22) and four were case series (9, 10, 23, 24).

According to the Joanna Briggs Institute (JBI) critical appraisal criteria, five case reports (18–22) presented a low risk of bias (score ≥7), whereas one case report (17) demonstrated a moderate risk of bias (score 5–6). All case series studies (9, 10, 23, 24) achieved scores ≥7, indicating a low risk of bias (**Supplementary Table 1**).

**Figure 2:**
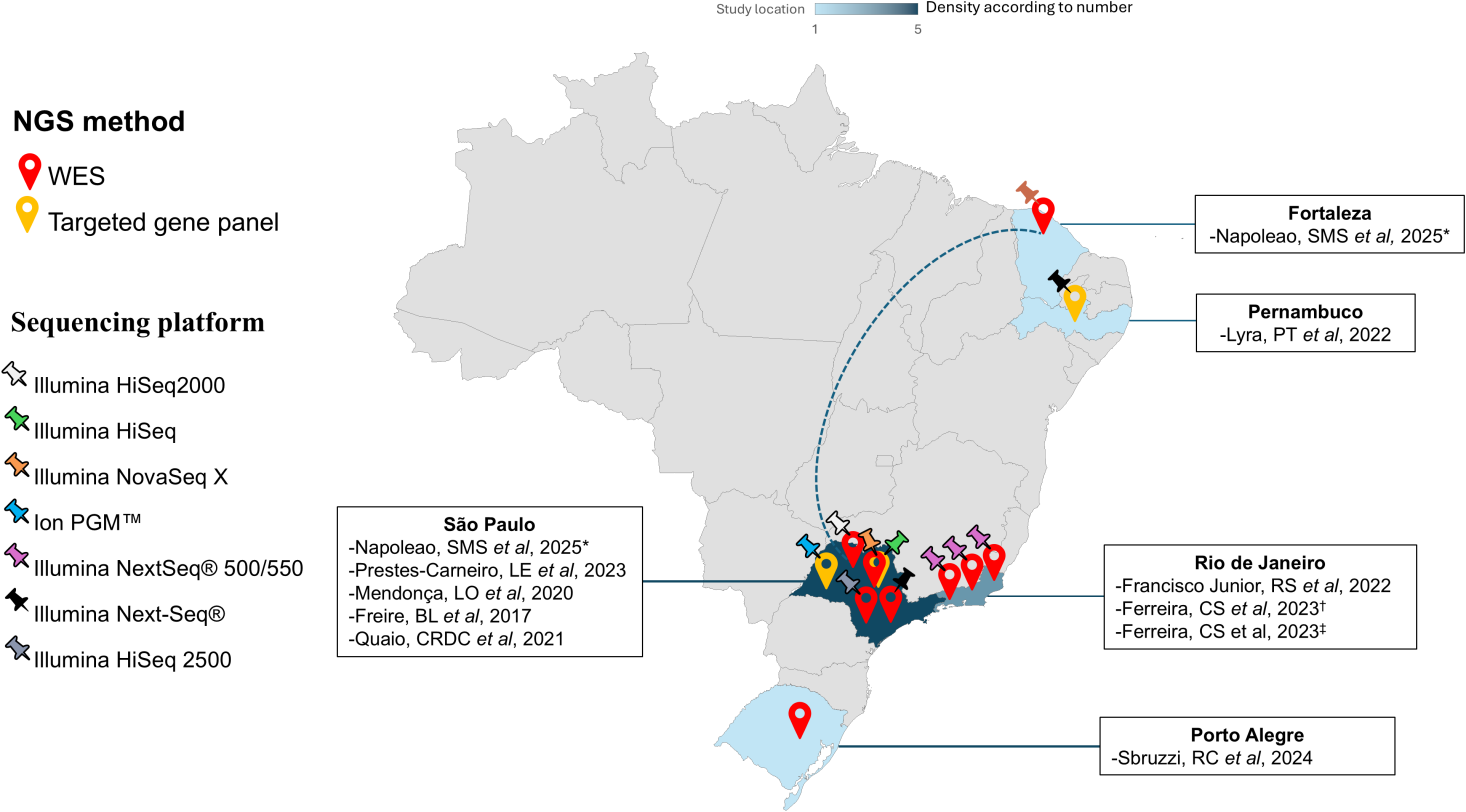
Geographic distribution of Brazilian studies included in the systematic review and their sequencing methods and platforms. The number of studies included is represented by the density in each Brazilian state. Map markers indicate the types of NGS methods used in each study, while map pins represent the sequencing platforms applied. *The study conducted by Napoleao, SMS et al, 2025 was carried out in two states (Fortaleza and São Paulo) and is represented by a dotted arch. ^†^Ferreira, CS et al, 2023 (9). ^‡^Ferreira, CS et al, 2023 (10).

Most studies were conducted in the states of Rio de Janeiro (n, 3) (9, 10, 20) and São Paulo (n, 4) (19, 21, 22, 24), both located in the Southeast region of Brazil. Additional studies were performed in Pernambuco (23), in the Northeast region, and Rio Grande do Sul (18), in the South region. One study (17) encompassed two cities — Fortaleza and São Paulo, located in the Northeast and Southeast regions, respectively. No studies were conducted in the North or Midwest regions. The geographic distribution of studies included is shown in **Figure 2**. Most patients resided in the same locality where the corresponding studies were carried out; however, four studies (17, 18, 21, 24) did not report patients’ places of residence (**Table 2**). A total of 419 patients were mentioned in the included studies, of whom 82 (19.6%) received a genetic diagnosis.

**Table 2:** Case series and case reports selected for systematic review.

First Author, Year	Patients’ location	Study location	Total case numbers (n)	NGS Method	Cases with genetic diagnosis n (%)*	IEI identified	Other clinical manifestations	History of Infections	Affected genes	Type of study
Napoleao, SMS et al, 2025 (17)	NR	Fortaleza e São Paulo	4	WES	2	ISG15 deficiency	Epilepsy, mild intellectual deficits, bilateral hearing loss and small intracranial calcifications; Encephalitis characterized by convulsive seizures and fever, as well as CGL, skin lesions, and lymph node abscesses	*Histoplasma* sp.infection, vulvo vaginitis, dermatophytosis, tuberculous lymphadenitis without mycobacterial isolation, tuberculosis by *Mycobacterium bovis*	*ISG15*	Case Report
Sbruzzi, RC et al, 2024 (18)	NR	Porto Alegre	4	WES	1	SCID	BCG-itis following BCG vaccination; Mixed chimerism post-HSCT	Recurrent infections (bacterial and viral); prolonged SARS-CoV-2 infection (positive test for 6 months)	*JAK3*	Case Report
Prestes-Carneiro, LE et al, 2023 (19)	Presidente Prudente	São Paulo	1	Targeted gene panel and WES	0	LOCID	Severe plaque psoriasis, psoriatic arthritis, Crohn’s disease	COVID-19 and dengue	NR/No variant disclosed^†^	Case Report
Francisco Junior, RS et al, 2022 (20)	Rio de Janeiro	Rio de Janeiro	2	WES	2	XLA	NR	Obliterative bronchiolitis, pneumonia, sepsis	*BTK* and *TGFβ1*	Case Report
Mendonça, LO et al, 2020 (21)	NR	São Paulo	1	Targeted gene panel	1	DIRA	Severe disabling osteomyelitis with mild pustular skin rash	NR	*IL1RN*	Case Report
Freire, BL et al, 2017 (22)	São Paulo	São Paulo	1	WES	1	Fanconi anemia-like syndrome	Heart disease, Microcephaly, Neuropsychomotor delay, Dysmorphisms, Subclinical cancer in mother	NR	*BRCA1*	Case Report
Ferreira, CS et al, 2023 (9)	Rio de Janeiro	Rio de Janeiro	20	WES	20 (100)	NR	NR	NR	*CARD9* ^‡^ *CFTR* *IFNG* *PSENEN* *RELA* *RNF31* *TNFRSF13B* *TNFRSF1A*	Case Series
Ferreira, CS et al, 2023 (10)	São Paulo	Rio de Janeiro	13	WES	13 (100)	XLA, XHIGM, BENTA, WAS, C6 deficiency, CVID8	NR	Sinusite, Pneumonia, *Pseudomonas* sp. Infection	*BTK, CD40LG, CARD11, WAS, CYBB, C6, LRBA, ABCA12, SLC25A13*	Case Series
Lyra, PT et al, 2022 (23)	Recife	Pernambuco	53	Targeted gene panel	7 (13.2)	CGD, MSMD, SCID	Higher frequency of systemic symptomatology	NR	*CYBB*, *STAT1*, *IL12B*, *IL12RB1*, *RAG1*, *CGD*	Case Series
Quaio, CRDC et al, 2021 (24)	NR	São Paulo	320	WES	35 (10.9)	SAG, FMF, CVD, C8D, SCID due to IL7R deficiency, Ataxia-telangiectasia, CD27 deficiency, CID due to GINS1 deficiency, IFN-γR1 deficiency, MKD, CD45 deficiency, BLS I	NR	NR	*RNASEH2B*, *CFTR*, *MEFV*, *TNFRSF13B*, *C8B*, *CTC1*, *FANCC*, *IL7R*, *ATM*, *C8A*, *CD27*, *FANCA*, *GINS1*, *IFNGR1*, *MVK*, *NBAS*, *PTPRC*, *RFXAP*, *SBDS*, *SMARCAL1*, *VPS13B* and *WRAP53*	Case Series

*Percentages were calculated only for case series studies. ^†^ Genetic testing with targeted gene panel was reported as negative for pathogenic/likely pathogenic variants, while no clinically relevant variants were detected with WES; however the manuscript does not detail variant prioritization and clinical interpretation criteria, or the complete list of variants identified. ^‡^ Data extracted from supplementary material. Abbreviations: Next generation sequencing (NGS), Inborn Errors of Immunity (IEI), Not Reported (NR), Whole Exome Sequencing (WES), Chronic Granulomatous Lymphadenitis (CGL), Severe Combined Immunodeficiency (SCID), Bacillus *Calmette-Guérin* (BCG), Hematopoietic Stem Cell Transplantation (HSCT), Severe Acute Respiratory Syndrome Coronavirus 2 (SARS-CoV-2), Late-onset Combined Immunodeficiency (LOCID), Coronavirus Disease 2019 (COVID-19), X-linked Agammaglobulinemia (XLA), Deficiency of Interleukin-1 Receptor Antagonist (DIRA), American College of Medical Genetics and Genomics and the Association (ACMG), X-linked Hyper-IgM Syndrome (XHIGM), B cell Expansion with NF-κB and T cell Anergy (BENTA), Wiskott-Aldrich Syndrome (WAS), Common Variable Immunodeficiency Type 8 with Autoimmunity (CVID8), Chronic Granulomatous Disease (CGD), Mendelian Susceptibility to Mycobacterial Disease (MSMD), Aicardi-Goutières syndrome (SAG), Familial Mediterranean Fever (FMF), Common Variable Immunodeficiency (CVID), Complement Component 8 deficiency (C8D), Combined Immunodeficiency (CID), Interferon-Gamma Receptor 1 (IFN-γR1), Mevalonate Kinase Deficiency (MKD), Bare Lymphocyte Syndrome Type II (BLS I).

### 3.2 NGS methods included in genetic diagnosis of IEI

**Figure 3:**
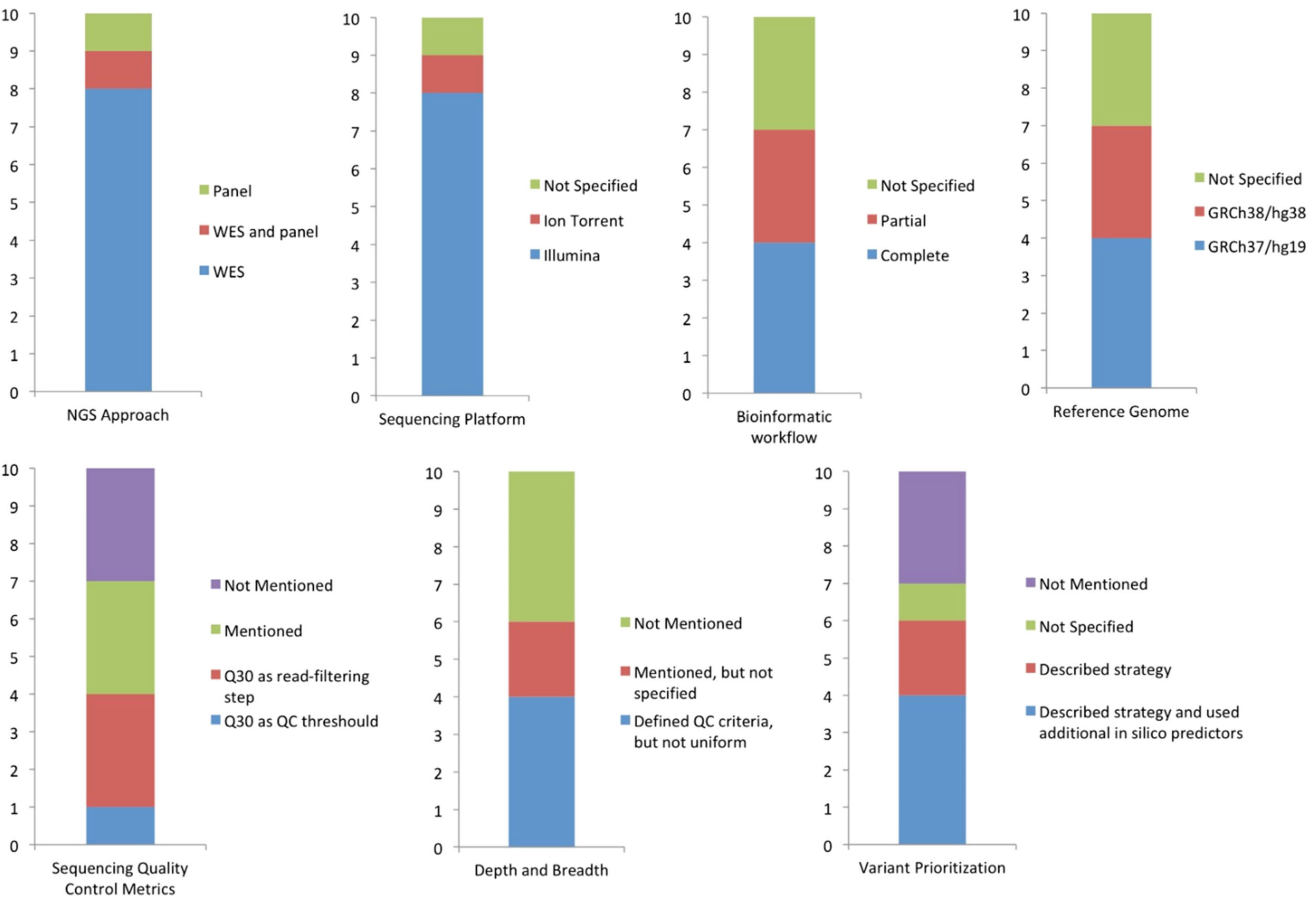
NGS methods, quality control assessment and variant prioritization detailing across the ten studies. WES: Whole exome sequencing. QC: quality control.

Most Brazilian studies employed WES as the primary molecular diagnostic approach (n, 7) (9, 10, 17, 18, 20, 22, 24). Two studies (21, 23) utilized targeted gene panels, and one study (19) applied both targeted panel sequencing and WES methodologies as shown in **Figure 3**. Notably, all studies performed NGS sequencing only in singleton (proband) with no evidence of trio analysis, and none reported the use of whole-genome sequencing (WGS).

The case report studies (17–22) collectively described thirteen patients, of whom seven obtained a confirmed genetic diagnosis (**Table 2**). Among the case series (9, 10, 23, 24), a total of 406 patients were included, with 75 receiving a molecular diagnosis. In the studies conducted by Ferreira and colleagues (9, 10), the use of WES enabled the identification of pathogenic variants in all patients, corresponding to a diagnostic yield of 100%. In contrast, Quaio et al. (24) achieved a genetic diagnosis in 35 (10.9%) of 320 patients with rare Mendelian disorders using WES. However, this study included not only IEI patients, but algo individuals with other diseases. Another low diagnostic yield (13.2%) was observed in the study by Lyra and colleagues (23), which employed targeted gene panel sequencing, and identified IEI causal variants in 7 of 53 individuals who experienced adverse events following BCG vaccination (**Table 2**).

### 3.3 IEI identified

The IEI reported in the case studies (17–22) comprised ISG15 deficiency, severe combined immunodeficiency (SCID), late-onset combined immunodeficiency (LOCID), X-linked agammaglobulinemia (XLA), deficiency of the interleukin-1 receptor antagonist (DIRA), and Fanconi anemia–like syndrome. In contrast, the case series (9, 10, 23, 24) demonstrated a high degree of heterogeneity in the spectrum of identified IEI, with SCID representing the most frequently reported condition (**Table 2**).

### 3.4 Infections and other clinical manifestations described among studies

A broad spectrum of clinical manifestations was described in the case report studies (17–22), encompassing neurological, dermatological, gastrointestinal, hematological, cardiovascular, and neoplastic conditions (**Table 2**). In contrast, the case series (9, 10, 23, 24) did not provide detailed descriptions of the clinical features observed in the included patients. The infections reported in the case reports (17–22) included fungal infections such as histoplasmosis and dermatophytosis; bacterial infections, including tuberculosis and recurrent bacterial infections; viral infections such as prolonged SARS-CoV-2 infection, COVID-19, and dengue; as well as respiratory complications, including pneumonia, bronchiolitis, sepsis, and systemic inflammatory responses. In the case series (10), only one study reported a history of infections including sinusitis, pneumonia, and *Pseudomonas* sp. Infection (**Table 2**).

### 3.5 IEI-associated genes identified across studies

**Figure 4:**
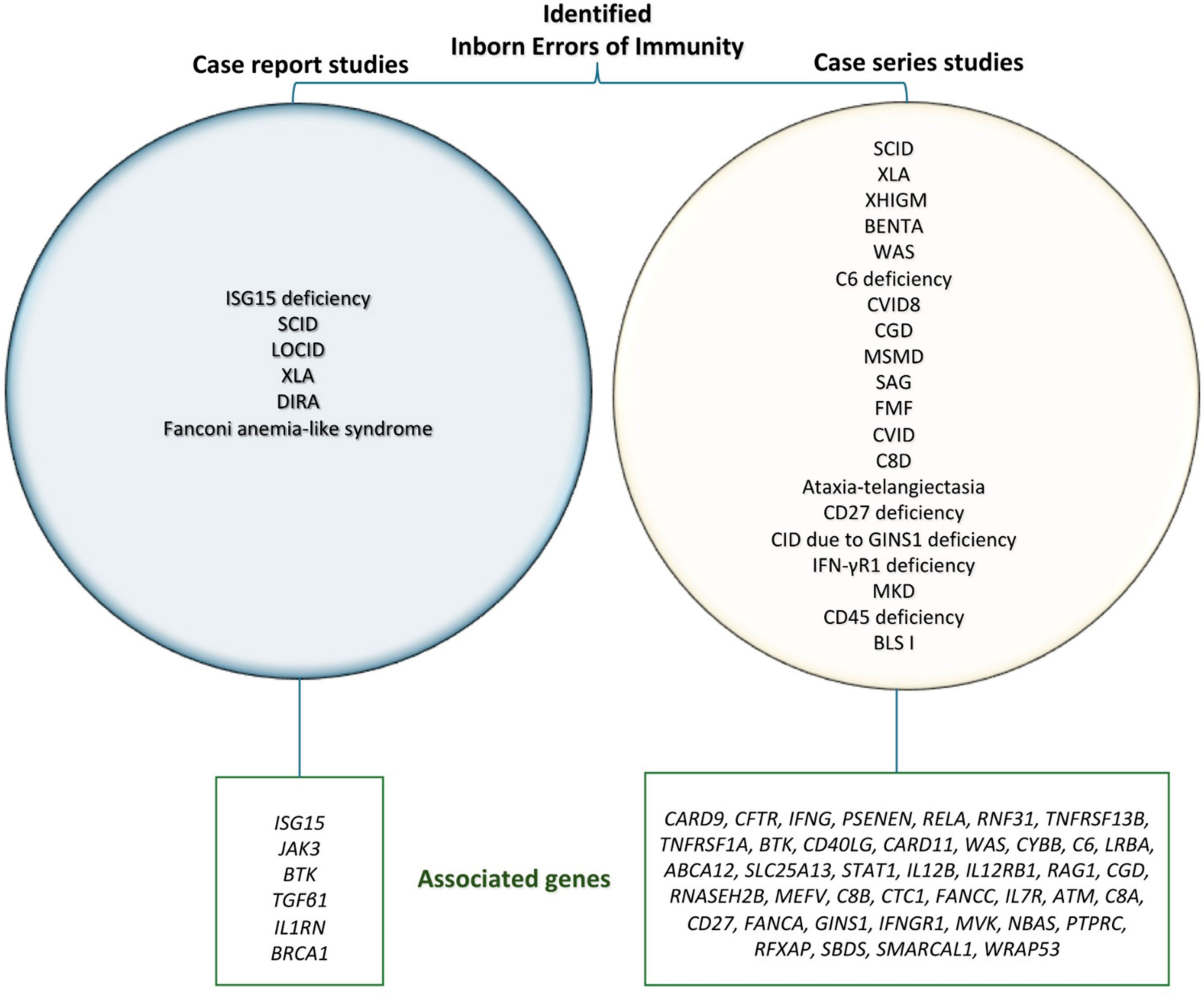
IEI genes with found variants of interest in the Brazilian studies. Variants in *BTK*, *CFTR*, *CYBB*, and *TNFRSF13B* were found in two different studies. All other genes were mentioned in only one study. Abbreviations: Severe Combined Immunodeficiency (SCID), X-linked Agammaglobulinemia (XLA), Late-onset Combined Immunodeficiency (LOCID), Deficiency of Interleukin-1 Receptor Antagonist (DIRA), X-linked Hyper-IgM Syndrome (XHIGM), B cell Expansion with NF-κB and T cell Anergy (BENTA), Wiskott-Aldrich Syndrome (WAS), Common Variable Immunodeficiency Type 8 with Autoimmunity (CVID8), Chronic Granulomatous Disease (CGD), Mendelian Susceptibility to Mycobacterial Disease (MSMD), Aicardi-Goutières syndrome (SAG), Familial Mediterranean Fever (FMF), Common Variable Immunodeficiency (CVID), Complement Component 8 deficiency (C8D), Combined Immunodeficiency (CID), Mevalonate Kinase Deficiency (MKD), Bare Lymphocyte Syndrome Type II (BLS I).

In the case report studies (17–22), NGS identified pathogenic or likely pathogenic variants in the *ISG15*, *JAK3*, *BTK*, *TGFβ1*, *IL1RN*, and *BRCA1* genes. Only one study did not identify any clinically significant genetic alterations. In the case series (9, 10, 23, 24), a broad range of affected genes was reported, including *BTK*, *CD40LG*, *CARD11*, *WAS*, *CYBB*, *C6*, *LRBA*, *STAT1*, *IL12B*, *IL12RB1*, *RAG1*, *CGD*, *RNASEH2B*, *CFTR*, *MEFV*, *TNFRSF13B*, *C8B*, *CTC1*, *FANCC*, *IL7R*, *ATM*, *C8A*, *CD27*, *FANCA*, *GINS1*, *IFNGR1*, *MVK*, *NBAS*, *PTPRC*, *RFXAP*, *SBDS*, *SMARCAL1*, *VPS13B*, and *WRAP53*. Furthermore, one study (9) reported 116 variants classified as rare pathogenic or likely pathogenic according to the American College of Medical Genetics and Genomics/Association for Molecular Pathology (ACMG/AMP) guidelines (**Figure 4**). Additionally, the study reported eight variants associated with IEI-disorders in heterozygosity in *CARD9*, *CFTR*, *IFNG*, *PSENEN*, *RELA*, *RNF31*, *TNFRSF13B*, and *TNFRSF1A* genes, all with a recessive inheritance pattern. However, no evidence of compound heterozygosity was found (**Table 2**).

### 3.6 Heterogeneity in NGS data processing workflows and variant analysis

Considerable heterogeneity was observed across studies regarding the level of methodological detail (**Table 1**). In several manuscripts, key information to assess analytical validity − such as sequencing quality metrics, data-processing workflows, and variant filtering and interpretation criteria – were incomplete or absent (**Figure 3**).

Although WES was the predominant approach (n, 8) (9, 10, 17–20, 22, 24), sequencing platforms varied. Illumina platforms (eg. NextSeq and HiSeq) were more frequently used, while one study (21) applied the Ion Torrent platform (with Ion PGM system) and another (18) did not specify the platform. Five studies (9, 10, 20, 23, 24) cited only the platform family, without naming the system model, and three of these (9, 10, 20) reported exclusively the sequencing reagent kit (Illumina NextSeq 500/550 High Output Kit v2). As this kit is compatible with different systems within the platform family, lack of explicit equipment identification prevents assessment of technical capabilities and compatibility with downstream analysis protocols.

Only four studies (9, 10, 20, 22) provided a complete bioinformatic workflow, including the software used for read alignment (plus the specific reference genome), post-alignment processing, variant calling, and annotation. Three studies (17, 23, 24) partially described the pipeline, and three (18, 19, 21) did not report any workflow details in the manuscript, supplementary material, or referenced documents. One study (21) cited an external methodological publication instead of describing the pipeline applied, although the cited reference includes more than one possible pipeline. Genome builds were inconsistently reported: four studies (17, 19, 22, 24) used GRCh37/hg19, three (9, 10, 20) used GRCh38/hg38, and the remaining studies did not specify the reference genome. Alignment tools included Bowtie2 and BWA-MEM, variant calling was most frequently performed with GATK, and annotation tools varied (ANNOVAR, VEP, SnpEff/SnpSift).

Reporting of sequencing quality control (QC) metrics was inconsistent, limiting between-study comparison and preventing objective evaluation of analytical reliability. Six studies (9, 10, 20, 22–24) mentioned QC metrics; however, in half of them (9, 10, 20), Q30 was cited only as a *read-filtering step*, rather than as a sequencing-quality metric. Only one study (23) reported Q30 as a predefined QC threshold (“≥90% bases that meet Q30”), consistent with clinical NGS requirements. Depth and/or breadth of coverage (e.g., ≥20× and percentage of bases ≥30×) were reported in two studies (21, 22) as descriptive sequencing results for patients, without stating whether those values met analytical thresholds. Four studies (9, 10, 23, 24) defined depth/breadth thresholds as QC criteria, but cutoff values were not uniform (coverage depth ≥20×; average exon depth ~120× and 92% bases ≥20×; or ≥95% bases ≥10×). Notably, none of the studies reported all essential QC metrics as predefined thresholds (*breadth of coverage, depth of coverage, and %Q30*). One study (23) reported additional QC metrics, such as uniformity of coverage and mapped-read percentage, reflecting a more comprehensive approach to data integrity and analytical reliability.

Most studies (9, 10, 18, 20, 22, 24) described strategies for variant prioritization and clinical interpretation, with four (10, 18, 20, 22) reporting the additional use of *in silico* pathogenicity predictors and minor allele frequency (MAF) filters. Only one study (22) utilized the ABraOM database to filter rare variants (MAF ≤ 0.1%) in patients with consanguinity confirmed by family pedigrees. One study (17) did not specify interpretation tools, and three (19, 21, 23) did not report how variants were analyzed. Filtering and prioritization criteria differed markedly, reflecting the heterogeneity and complexity of IEI that require a patient-specific approach, although all studies that reported variant interpretation adopted ACMG/AMP guidelines for classification.

In summary, none of the manuscripts provided a fully traceable NGS workflow encompassing sequencing platform, QC thresholds, variant calling, annotation, filtering, and clinical interpretation.

## 4 Discussion

The implementation of NGS technologies has transformed the diagnosis of IEI worldwide, enabling the identification of causative variants and guiding precision treatment strategies. This study systematically reviewed the literature reporting Brazilian studies to evaluate the applications of these technologies in the national context, seeking to understand the clinical and methodological characteristics of these studies. Our systematic review collected ten Brazilian studies that use NGS for the diagnosis of IEI, revealing that the studies are still limited and heterogeneous in terms of design, methodology, and reporting standards. This heterogeneity reflects both the technological diversity of sequencing platforms and the lack of standardized guidelines to reporting studies’ findings.

In Brazil, patients within the private healthcare sector have greater access to advanced diagnostic testing and are predominantly concentrated in the Southeastern region. Since 2021, WES has been incorporated into private health insurance coverage. The state of São Paulo concentrates some of the most advanced genetic laboratories in Brazil, with a broad portfolio of genetic tests for the private sector (27). In stark contrast, public access to genetic diagnosis for rare diseases began in 2014. Regarding IEI, a more pronounced gap still stands as access to NGS in the public scenario remains mostly associated with the context of research projects. Moreover, implementing exome and WGS in the SUS remains challenging due to sophisticated infrastructure needs, advanced technology, specialized personnel for data analysis, complex regulations, high costs, and infrastructure constraints (28). Overall, WES use has increased nationally and represents most genetic tests in specialized labs for complex disorders. Health infrastructure disparities, regional socioeconomic differences, specialist shortages, and genetic variant interpretation complexity contribute to variable NGS diagnostic rates across Brazil. Earlier diagnosis and better regional infrastructure correlate with higher success rates (28).

In this review, most of the included studies were conducted in large medical centers in the states of Rio de Janeiro and São Paulo, located in the southeastern region of Brazil. These centers offer referral services for rare diseases accredited by the Ministry of Health (9, 10). However, many of them face challenges in implementing a broad portfolio of genetic tests, especially for the diagnosis of IEI, and the SUS does not reimburse diagnostic tests for most genetic diseases (9, 10, 29). Therefore, a large portion of the Brazilian population (approximately 75%) that depends exclusively on the SUS has limited access to comprehensive genetic diagnosis and treatment (30). This situation leads to significant regional and social inequities that impact healthcare access, resulting in a complementary dual system. Importantly, this geographic gap represents not only a limitation in research output, but also a critical clinical concern. Delayed access to NGS-based diagnosis in underserved regions may postpone definitive diagnosis and, consequently, access to life-saving interventions such as hematopoietic stem cell transplantation (HSCT). This is particularly relevant for severe conditions like SCID, in which early molecular diagnosis is essential to guide timely transplantation and improve survival outcomes. Early transplantation, ideally performed within the first months of life and prior to the development of active infections, is associated with improved overall survival. Newborn screening for SCID has been fundamental in enabling early diagnosis and transplantation under optimal, infection-free conditions, increasing survival rates to over 90% in patients treated early (31). In contrast, delayed diagnosis and transplantation after the first year of life are associated with increased risks due to a higher incidence of infections and complications. In addition, delayed diagnosis may contribute to prolonged exposure to severe or persistent infections, including viral infections such as SARS-CoV-2, further increasing morbidity and mortality in these patients, and also leading to a higher burden for the health system (32).

### 4.1 Heterogeneity of IEI diagnoses and clinical manifestations in Brazilian NGS studies

The IEI identified across studies were heterogeneous, with SCID and XLA being the most frequently reported conditions. Other diagnoses included LOCID, DIRA, ISG15 deficiency, and Fanconi anemia-like syndromes, demonstrating the broad phenotypic spectrum captured through NGS. This heterogeneity reflects the complex and multifactorial nature of IEI. The predominance of SCID and XLA across studies aligns with their established clinical significance and prevalence, respectively. SCID encompasses a spectrum of molecularly distinct subtypes that critically influence patient prognosis and therapeutic strategies, thereby underscoring the imperative for early and precise molecular diagnosis — an approach currently being integrated into Brazil’s neonatal screening protocols (33–35). On another hand, XLA displays substantial genetic variability within the Brazilian population, but most of cases are only biochemically diagnosed, accentuating the necessity of molecular confirmation to improve clinical management and genetic counseling (8, 10).

The clinical manifestations reported were equally diverse and included infectious complications, such as pneumonia, sinusitis, tuberculosis, and viral infections (including prolonged COVID-19 and dengue), as well as autoimmune and inflammatory features. These findings align closely with the broad clinical spectrum described in the wider IEI literature, where recurrent and severe infections predominantly affecting the respiratory and systemic systems are hallmark presentations (36, 37). The susceptibility to a range of bacterial, mycobacterial, and viral pathogens — including neglected tropical infections like arboviruses — reflects the fundamental immunological deficits characteristic of IEIs (38–40).

Globally, diagnostic yield for IEI using NGS is around 29% (range 10-70%), reaching 56% with targeted panels and 70% with WES. In contrast, the Brazilian studies included in this review showed marked variability, with diagnostic rates ranging from 10% to 100%. This discrepancy likely reflects differences in cohort selection, with higher yields observed in highly selected cases with strong clinical suspicion, suggesting potential selection bias.

Furthermore, autoimmune and autoinflammatory manifestations are increasingly recognized as significant components of IEI phenotypes beyond recurrent infections, mirroring observations in global cohorts where immune dysregulation pathways play a pivotal role (41, 42). Thus, the diverse clinical manifestations documented in Brazilian studies not only corroborate international data, but also emphasize the necessity for heightened awareness and comprehensive diagnostic evaluations incorporating genetic insights to improve patient outcomes in diverse clinical contexts.

### 4.2 International perspectives on the use of NGS for IEI diagnosis

Overall, this review showed that the success rate of genetic diagnostic varied widely among studies, from 10.9% in large cohorts to 100% in small targeted series, reflecting differences in patient selection, sequencing methods, and variant interpretation pipelines. This variability reinforces the need for national standardization of analytical criteria to ensure reproducibility and comparability of results. Although many studies focus on diagnostic methods, some explicitly detail how exome, genome, and gene panel sequencing are implemented in public health systems of other countries, especially in resource-limited contexts.

In European and US public health systems, WES and targeted gene panels are routinely implemented with standardized workflows (43, 44). In the Netherlands’ public healthcare framework, routine WES is established practice for undiagnosed primary immunodeficiencies (PID) using uniform bioinformatics pipelines (43). Italian National Health Service centers employ custom PID-targeted NGS panels for complex immunodeficiencies (45), while US National Institutes of Health (NIH)-funded consortia utilize WES and comprehensive PID panels through systematic genomic approaches (46). This international consistency in WES/panel utilization provides a benchmark for standardizing NGS protocols within Brazil’s SUS infrastructure.

Regarding Low- and Middle-Income Countries, in a study from South Africa, WES and targeted gene panels were applied to patients from the public healthcare system (47). The study reported that, despite technological progress, access to WES/WGS remains restricted to research settings due to high costs and analytical complexity. Gene panels are the main clinical diagnostic tool available in the public system, while WES/WGS are primarily used in research projects. Funding for genetic testing is limited, with some tests covered by pilot programs or external partnerships. The clinical impact is enormous: 67% of patients with a molecular diagnosis had changes in clinical management, and diagnosed families received adequate genetic counseling (47).

In Egypt, the diagnostic strategy is adapted to the availability of resources in the public system. A study highlights a stepwise use of Sanger sequencing, NGS panels, and WES, choosing the method according to the patient’s phenotype and financial feasibility. This approach enabled diagnosis in 56% of cases, demonstrating that the integration of different methods is essential in public systems with limited resources (48).

In countries such as South Korea, Malaysia, and Turkey, genetic sequencing is performed in university hospitals or public reference centers, with direct impact on diagnosis and treatment, but details regarding funding and universal access vary and are not always explicitly discussed in studies. In South Korea, clinical exome sequencing in public tertiary hospitals resulted in diagnosis in 40.5% of cases, identifying pathogenic, including novel, variants with therapeutic implications (49).

In Turkey, large-scale WES in public centers yielded a probable diagnosis in 41.1% of patients, with discovery of novel variants and new candidate genes, showing the value of collaboration among centers and the importance of expanded access to sequencing (50). In Malaysia, a pilot study in a public hospital showed that WES enabled diagnosis in 46.7% of pediatric cases, altering clinical management and enabling more appropriate therapies (14).

### 4.3 Lack of standardization and reproducibility in NGS analyses

One of the main limitations identified among Brazilian studies employing NGS for the diagnosis of IEI is the absence of methodological standardization across laboratories. Most reports relied on different sequencing platforms, variant-calling pipelines and annotation tools, which hampers reproducibility and prevents meaningful comparison of diagnostic yield. While commercial kits and standardized pipelines are common in reference centers, other groups still use research-oriented or in-house workflows, frequently without harmonized analytical validation steps.

Essential sequencing quality parameters — including thresholds that ensure adequate coverage for reliable variant detection — are often missing or inconsistently reported. This absence further compromises data reproducibility because variant-calling confidence is directly influenced by depth and uniformity of coverage, which in turn depend on the sequencing platform and assay design (singleton vs. trio). Establishing minimum analytical standards for NGS-based clinical interpretation would enable comparability across studies, facilitate data integration, support multicenter analyses and shared variant repositories — critical steps for national implementation of precision medicine.

#### 4.3.1 Recommendations for clinical NGS

According to ACMG/AMP technical standards for NGS, clinical laboratories must predefine and report analytical quality metrics to ensure reliability of variant detection and support clinical interpretation (51, 52). To meet the guidelines requirements and mitigate the methodological variability observed in the included studies, we propose that future publications and laboratories report, at a minimum, the following QC parameters: (i) base quality — % of bases ≥ Q30 (Phred-scale); (ii) Mean depth of coverage (reads over the target region); (iii) breadth of coverage — % of bases in target region that reach or exceed a specified depth threshold.

Importantly, the definition of “target region” depends on the sequencing strategy. Notably, inconsistent use of terminology was observed across studies. For example, one targeted gene panel study (23) claims “whole genome coverage” despite sequencing being restricted to a predefined set of genes. Such discrepancies illustrate how imprecise definition of target regions may lead to overestimation of sequencing performance and hinder cross-study comparability, reinforcing the need for standardized NGS reporting practices. For WGS, the target region corresponds to the entire genome (although coverage is often assessed over clinically interpretable regions, such as the entire exome or ACMG/AMP 59 genes) (53); for WES, it corresponds only to captured exons; and for targeted gene panels, it includes only predefined genes or exons.

Additional QC metrics are required depending on the sequencing strategy. WGS should report (iv) coverage uniformity — % of bases within a defined percentage of the mean coverage depth, and (v) mapping rate — % of reads aligned to the reference genome. WES and targeted panels require explicit assessment of capture efficiency through (vi) on-target rate — % of aligned bases within the target region. Collectively, these metrics allow evaluation of whether the sequencing data meets analytical requirements for confident variant calling and clinical interpretation.

ACMG/AMP guidelines also require laboratories to define a minimum coverage threshold for variant calling. Because heterozygous variants are expected to appear in ~50% of read observations, insufficient depth increases the risk of incorrect zygosity assignment and false negatives. ACMG/AMP recommends a minimum of 10–20× per base within the target region. At 10× depth, the probability of detecting the alternate allele at least once is ~99.9%, whereas 20× reduces the probability of missing it to ~0.0001% (~99.9999% detection confidence). For this reason, 20× is widely adopted as the minimum per-base depth threshold for clinical interpretation.

Consequently, breadth of coverage, defined as the percentage of target bases achieving ≥20× depth, has become a critical QC metric for reporting sequencing adequacy, especially for WES and WGS. Currently, it is recommended that WES achieve a mean depth of ~100× with 90–95% of target bases at ≥10–20× (which may be reduced to ~70× in trio sequencing), while WGS typically requires ≥30× mean depth (54, 55). Notably, (56) demonstrated that WES reaches ~95% SNP-detection sensitivity at ~40× mean depth, whereas WGS achieves similar sensitivity at only ~14×, highlighting the importance of modality-specific QC thresholds (56).

In addition to reporting analytical quality metrics, ACMG/AMP technical standards emphasize that laboratories must provide a transparent description of the variant filtering and assessment strategy used. This includes disclosing filtering criteria (e.g., allele-frequency thresholds, inheritance mode, pathogenicity predictors, zygosity, and genomic context), as well as the software and databases used for annotation and prioritization. Importantly, regardless of the filtering approach, every variant identified in a disease-targeted test must undergo formal clinical interpretation following ACMG/AMP guidelines and be assigned a classification, along with the evidence supporting that classification. Transparent reporting of variant filtering logic and ACMG/AMP classification criteria enables reproducibility, prevents biased prioritization, and ensures that clinical conclusions are based on interpretable and auditable evidence.

However, analytical and bioinformatic rigor alone may not be sufficient for definitive variant classification. A major limitation identified across the included studies was the lack of integration between NGS findings and functional validation assays upon detection of VUS. According to ACMG/AMP guidelines, functional evidence is often required to support variant reclassification, particularly from VUS to actionable classifications (Likely Pathogenic and Pathogenic). The absence of such data may compromise the accuracy of genotype–phenotype correlations and limit definitive clinical diagnoses.

It is acknowledged that WES provides higher genomic resolution compared to Sanger sequencing or targeted panel NGS, which is inherently associated with an increased detection rate of VUS, given the expanded number of genes interrogated. This challenge is particularly pronounced in the Brazilian population, which remains underrepresented in publicly available genomic reference databases, directly impacting variant interpretation and contributing to disproportionately higher VUS rates compared to well-characterized populations (27, 57).

Trio-based WES (proband and both biological parents) improves variant interpretation through segregation analysis, identifying compound heterozygous and *de novo* variants, narrowing down the number of VUS of interest, thus reducing manual curation burden, enhancing diagnostic yield and shortening analysis turnaround time. VUS stratification by evidence levels, routine family assessment, enhanced genetic counseling, laboratory evidence integration, and population diversity in genomic databases further aids reclassification according to ACMG/AMP guidelines. However, our findings show Brazilian studies still lack standardization in dealing with VUS. Indeed, this is another gap the RENOMIEII was built to tackle. Moreover, elevated WES costs — particularly trio analysis — still represent a significant barrier to widespread implementation in Brazil, limiting private health insurance reimbursement and access (27, 57).

### 4.4 Limited ancestry data and implications for variant interpretation

Another major challenge identified in Brazilian genomic studies on IEI is the lack of ancestry information for the studied cohorts (9, 10, 17–24). The absence of genomic ancestry analyses has important implications for the interpretation of sequencing results. Because the majority of reference databases used for variant filtering and pathogenicity prediction, such as gnomAD, 1000 Genomes, or ClinVar, are composed predominantly of individuals of European or East Asian ancestry, variants that are common in underrepresented populations may be incorrectly classified as rare and therefore gain PM2 criteria which may increase ACGM classification points towards the pathogenic direction in a misleading way (12, 58). Most of the studies included in this systematic review did not use Brazilian allele frequency databases in the analyses. Only one study reported to have used ABraOM to filter rare variants in consanguineous cases (22).

This lack of representation contributes to a higher rate of VUS and limits clinical decision-making. Incorporating local genomic data and ancestry-informed variant frequency references is therefore essential to improve diagnostic accuracy and reduce interpretation bias, particularly in a country with one of the world’s most genetically diverse populations (58). As we have now two available databases to check for allele frequency in the Brazilian population (namelly AbraOm and DNA do Brasil), moving forward we propose that all studies and clinical diagnostic exams make use of these tools when analyzing allele frequency of variants found in a Brazilian individual (ref Abraom e DNA do Brasil). Moreover, although having these references is a huge advance, as collectively these two databases account for less than five thousand individuals, it is also clear that we need to invest in sequencing many more Brazilians with proper geographical and ethnicity representation to enlarge our national database, hence continuing to statistically improve Brazilian allele frequency representation.

### 4.5 NGS as an exploratory strategy for discovering novel disease mechanisms

Beyond its diagnostic utility, NGS also serves as a powerful exploratory approach to uncover novel genetic mechanisms underlying immune dysregulation. In a recent study on multisystem inflammatory syndrome in children (MIS-C), a new disease associated with SARS-CoV-2, we (59) used WES to identify variants in genes related to interferon signaling and inflammasome activation, suggesting that host genetic background may modulate disease severity (59).

Such studies illustrate the potential of NGS to advance understanding of complex or emerging immune disorders, complementing its role in identifying known pathogenic variants (59). Expanding exploratory NGS applications in Brazil could foster the discovery of new immune pathways, improve disease classification, and reveal population-specific variants relevant to IEIs.

Brazilian initiatives have dedicated significant efforts to the early diagnosis and personalized management of patients with IEI (9, 10, 59). Collaborative genomic networks have emerged, fostering the standardization of genetic diagnostic protocols, sequencing procedures, and variant interpretation. Such efforts have enhanced consistency across centers and reduced diagnostic delays. Moreover, these collaborative networks have expanded access to advanced genomic technologies for patients across diverse regions, helping to mitigate geographic and social disparities within Brazil. Consequently, these initiatives have improved diagnostic accuracy, democratized access to genomic technologies, and contributed to better quality of life for patients and their families. In the near future, further advances are expected following the Ministry of Health’s recent announcement of a nationwide program to implement WES as a routine diagnostic test for SUS patients with rare diseases.

## 5 Conclusions

This systematic review provides an overview of NGS applications in IEI in Brazil. Despite recent advances, NGS remains concentrated in a few specialized centers, primarily in the Southeast region, with WES as the most frequently used approach and no reports of WGS implementation. The studies revealed a heterogeneous diagnostic yield and methodological diversity, reflecting differences in patient selection, sequencing platforms, and variant interpretation pipelines. SCID and XLA were the most frequently identified IEI, often associated with recurrent bacterial and viral infections. These findings underscore the need for national standardization of genomic analyses, inclusion of ancestry-informed approaches, and expansion of NGS access through collaborative and public health initiatives to achieve equitable and precise diagnosis of IEI across Brazil.

## Data Availability

The original contributions presented in the study are included in the article/**Supplementary Material**. Further inquiries can be directed to the corresponding author.
