# Supplementary material for: Overview of next-generation sequencing to the molecular diagnosis of inborn errors of immunity in Brazil: a systematic review
Source: Front Immunol. 2026 May 15;17:1794921. doi: 10.3389/fimmu.2026.1794921 (PMC13219264; doi:10.3389/fimmu.2026.1794921)
Supplement: Supplementary file 1 [file Table1.docx]

Supplementary Material

|  | **Authors and year** | **Q1** | **Q2** | **Q3** | **Q4** | **Q5** | **Q6** | **Q7** | **Q8** | **Q9** | **Q10** | **Total score** |
| --- | --- | --- | --- | --- | --- | --- | --- | --- | --- | --- | --- | --- |
| **Case Report** | Napoleao, SMS *et al,* 2025 (20) | Y | Y | Y | Y | Y | U | U | Y | - | - | 6 |
|  | Sbruzzi, RC *et al,* 2024 (25) | Y | Y | Y | Y | Y | Y | Y | Y | - | - | 8 |
|  | Prestes-Carneiro, LE *et al*, 2023 (19) | Y | Y | Y | Y | Y | Y | Y | Y | - | - | 8 |
|  | Francisco Junior, RS *et al,* 2022 (23) | Y | Y | Y | Y | Y | Y | Y | Y | - | - | 8 |
|  | Mendonça, LO *et al,* 2020 (22) | Y | Y | Y | Y | Y | Y | Y | Y | - | - | 8 |
|  | Freire, BL *et al,* 2017 (21) | Y | Y | Y | Y | Y | Y | Y | Y | - | - | 8 |
| **Case Series** | Quaio, CRDC *et al,* 2021 (18) | Y | Y | Y | Y | Y | N | Y | Y | Y | Y | 9 |
|  | Lyra, Paula T *et al*, 2022 (24) | Y | Y | Y | Y | Y | Y | Y | Y | Y | Y | 10 |
|  | Ferreira, CS *et al*, 2023 (9) | Y | Y | Y | Y | Y | N | N | Y | N | Y | 7 |
|  | Ferreira, CS *et al,* 2023 (10) | Y | Y | Y | Y | Y | Y | Y | N | N | Y | 8 |

**Table S1.** Assessment of the risk of bias among the studies included in the systematic review according to Joanna Briggs Institute Critical Appraisal Checklist

Joanna Briggs Institute Critical Appraisal Checklist for Case Report and Case Series. Case Report Questions: Q1 = Were patient’s demographic characteristics clearly described? Q2 = Was the patient’s history clearly described and presented as a timeline? Q3 = Was the current clinical condition of the patient on presentation clearly described? Q4 = Were diagnostic tests or assessment methods and the results clearly described? Q5 = Was the intervention(s) or treatment procedure(s) clearly described? Q6 = Was the post-intervention clinical condition clearly described? Q7 = Were adverse events (harms) or unanticipated events identified and described? Q8 = Does the case report provide takeaway lessons? Case Series Questions: Q1 = Were there clear criteria for inclusion in the case series? Q2 = Was the condition measured in a standard, reliable way for all participants included in the case series? Q3 = Were valid methods used for identification of the condition for all participants included in the case series? Q4 = Did the case series have consecutive inclusion of participants? Q5 = Did the case series have complete inclusion of participants? Q6 = Was there clear reporting of the demographics of the participants in the study? Q7 = Was there clear reporting of clinical information of the participants? Q8 = Were the outcomes or follow up results of cases clearly reported? Q9 = Was there clear reporting of the presenting site(s)/clinic(s) demographic information? Q10 = Was statistical analysis appropriate? Y = Yes; N = No; U = Unclear.
